# Effect of neridronate on axial involvement in patients with spondyloarthritis when biologics are not possible. Results of a monocentric study

**DOI:** 10.3389/fmed.2023.1282169

**Published:** 2023-11-20

**Authors:** Chiara Crotti, Raffaele Di Taranto, Francesco Orsini, Matteo Ferrito, Massimo Varenna, Ennio Giulio Favalli, Roberto Caporali

**Affiliations:** ^1^Bone Disease Unit, Department of Rheumatology and Medical Sciences, ASST G. Pini-CTO, Milan, Italy; ^2^Rheumatology Clinic, Department of Rheumatology and Medical Sciences, ASST G. Pini-CTO, Milan, Italy; ^3^Department of Clinical Sciences and Community Health, University of Milan, Milan, Italy

**Keywords:** spondyloarthropathy, sacroiliitis, Tumor necrosing factor alpha, disease activity score, bisphonates

## Abstract

**Introduction:**

This study aims to examine the potential effectiveness of intravenous neridronate (IVNer) on axial involvement in patients with spondyloarthritis (SpA) refractory to non-steroidal anti-inflammatory drugs (NSAIDs) but not eligible for biological disease-modifying antirheumatic drugs (bDMARDs).

**Method:**

Patients with active SpA (BASDAI score ≥ 4) and active sacroiliitis (SI) on MRI (according to ASAS MRI definition), who were NSAID-insufficient responder/intolerant but not eligible for bDMARDs, were retrospectively recruited in a tertiary rheumatology centre between September 2015 and December 2021. IVNer (100 mg) was administered to the patients on days 1, 4, 7, and 10. Responses were evaluated 60 days after the last infusion as the median changes from the baseline of BASDAI and Visual Analogue Scale (VAS) pain and there are improvements on MRI signs.

**Results:**

A total of 38 patients (26 axial SpA, 3 enteropathic arthritis, and 9 axial psoriatic arthritis) were included [66% women, mean age ± SD: 38.0 ± 14.1 years, mean disease duration: 30.5 ± 49.5 months (range 1.0–298), 47% HLAB27+]. The reason for bDMARD ineligibility was concurrent solid tumors (*n* = 6) or hematological (*n* = 1) malignancy, comorbidities (*n* = 11), or patient preference (*n* = 20). Both median BASDAI [5.83 (4.2–8.33) versus 3.66 (1.1–6.85), *p* < 0.001] and VAS pain [7 (5.75–8.0) versus 3 (1.0–7.0), *p* < 0.0001] significantly decreased after IVNer. Of 28 available MRI at follow-up, we observed a complete (36%) or partial (39%) resolution of sacroiliitis or a persistent activity (25%).

**Discussion:**

IVNer was effective in improving axial involvement in patients with SpA refractory to NSAIDs but not eligible for bDMARDs. IVNer can be considered as a potential alternative therapeutic option in selected settings.

## 1 Introduction

Sacroiliitis (SI) is a common clinical feature in spondyloarthritis (SpA) (1). The early involvement of sacroiliac joint (SIJ) had been challenging since the introduction of MRI because plain radiography shows only suspicious elements in grades I-II and irreversible issues in the later stages (2). In recent times, MRI can detect early modification of the subchondral bone and synovium, and the Assessment of SpondyloArthritis international Society (ASAS) MRI working group generated a consensus update and validation on standardized definitions for MRI lesions in the SIJ of patients with SpA (3). According to the 2022 ASAS-EULAR recommendations for the treatment of SpA (4), non-steroidal anti-inflammatory drugs (NSAIDs) should be the first-line therapy, with two consecutive courses of maximized regimen in non-responder patients. In patients who failed or were intolerant to NSAIDs, biologic disease-modifying anti-rheumatic drugs (bDMARDs) as tumor necrosis factors-alfa (TNF-*α*) or interleukin-17 (IL-17) inhibitors are the first suggested option. However, in rheumatological settings, the prescription of bDMARDs may not be appropriate due to absolute or relative contraindications to the use of this drug class in a variable percentage of patients (<10%), which differs according to the reason of contraindication, i.e., comorbidities and malignancies (5, 6). In this scenario, the proper strategy to adopt is still debatable and represents a major challenge for rheumatologists, without any suggestion from the main international recommendations.

Bisphosphonates (BPs) demonstrated anti-inflammatory effects *in vitro* and *in vivo* (7, 8). Nitrogen-derived bisphosphonates (N-BPs) have been tested for their anti-inflammatory effects in the treatment of chronic arthritis, with variable outcomes (9). While no significant improvement was reported in the therapy of rheumatoid arthritis (10), pamidronate improved clinimetric (11) and radiologic parameters (12) in patients with SpA. Furthermore, N-BPs have been demonstrated to reduce the level of pro-inflammatory cytokines [i.e., tumor necrosis factor-*α* (TNF-*α*), interleukin-1 (IL-1), and IL-6 (8, 13). Recent studies have demonstrated immune-modulating effects of oral N-BPs in patients treated for osteoporosis compared to those with osteopenia and healthy controls (14). In Italy, neridronate is registered for treating several conditions including osteogenesis imperfecta (2 mg/kg IV every 3 months], Paget’s disease of bone (100 mg IV two consecutive days), and complex regional pain syndrome (CRPS) (100 mg IV every 3 days, for four infusions).

We aimed to investigate the clinical and MRI response after IV neridronate (IVNer) in patients affected by SpA with axial involvement refractory to NSAIDs but not eligible for a second-line therapy with bDMARDs.

## 2 Methods

### 2.1 Study population

For our study, we retrospectively included patients who were 18 years or older and affected by active SpA (as defined by a BASDAI score ≥ 4) with axial involvement [active sacroiliitis (SI) on MRI according to ASAS MRI definition] (15). These patients had failed or were intolerant to NSAIDs but not eligible for bDMARDs. They were treated with IVNer between September 2015 and December 2021.

Ineligibility criteria for bDMARDs were the presence of absolute contraindications (i.e. solid tumor, precancerous lesions, comorbidities), chronic infections without appropriate prophylaxis or frequent serious infections. Additionally, individuals with relative contraindications such as patient’s preference (i.e., unwillingness to start an immunosuppressive treatment) were also excluded.

All patients gave written informed consent. All analyzed clinical information were reported as anonymous aggregate data, excluding any identifiable medical information.

### 2.2 Treatment

IVNer (100 mg over 3 h in 500 mL of 0.9% saline solution) was administered on days 1, 4, 7, and 10 in an outpatient facility. During the first visit, Visual Analogue Scale (VAS) pain score and BASDAI score were collected, and ESR and CRP values were reported. IVNer-response was evaluated after 60 days since the last infusion as the mean changes from baseline for BASDAI score, VAS pain score, ESR and CRP values, and the improvement on MRI signs [i.e., resolution of bone marrow edema (BME) at SIJ]. A clinical response was rated as an improvement of ≥2 cm in VAS pain score (16).

### 2.3 Statistical analysis

Statistical analysis was performed using SPSS Statistic Editor (IBM software, Version 28.0.1.1 (14)). The results were presented as mean ± DS or median (IQR). The normality test was performed. Continuous variables were compared using the T-test for unpaired data, and the Mann–Whitney U test was used when the normality assumption could not be confirmed. Changes from baseline were analyzed by non-parametric statistical hypothesis Wilcoxon signed-rank test, for repetitive variables. Bivariate analysis was performed using the Pearson *χ*^2^ test. Logistic regression was used to find the possible predictors for treatment success. The significance level was set at a value of *p* of less than 0.05.

## 3 Results

The screening process included 47 patients affected by sacroiliitis in SpA. Due to different etiologies of SI, seven patients with juvenile idiopathic arthritis-enthesitis related arthritis, one patient with Behçet’s disease, and one patient with aankylosing spondylitis (AS) with concomitant Paget disease of the sacrum and ileum were excluded from the study. A total of 25 (66%) patients were women with a mean age of 38.0 years ±14.1 on therapy. The mean disease duration at therapy was 30.5 ± 49.5 months (1.0–298). A total of 26 (68%) patients were affected by axSpA, 3 (8%) by inflammatory bowel disease-associated SpA, and 9 (24%) by psoriatic arthritis.

Out of all the patients, twenty three of them had (60%) presented exclusively the axial disease, nine (24%) had a predominantly axial disease with peripheral involvement (predominantly at the lower limbs), and six (16%) had a predominantly axial disease associated with entheseal inflammation. Associated fibromyalgia was present in six patients (16%). HLA-B27 was present in 18 (47%) patients, and MRI sacroiliac joint inflammation was unilateral in 19 (50%) patients. SIJ erosions were already present in 11 (29%) patients at MRI imaging. Patients’ characteristics are described in Table 1. Fourteen patients were not eligible for bDMARDs due to the concomitant solid tumor and relative or absolute morbidity (six solid tumors, one chronic myeloid leukemia; seven previous serious infections, two cervix HPV infection with H-SIL, one BK latent infection, and one chronic viral hepatitis). A total of 20 patients (52%) exhibited unwillingness to start immunosuppressive therapy with bDMARDs for fear of side effects, personal opinion, and impossibility to attend regular visits at hospital. Only one patient has been receiving sulphasalazine at a stable dosage for 3 months before the study entry, and it was maintained constant during the study.

**Table 1:** Patient demographics and baseline characteristics (*N* = 38).

Age, median (IQR), years	34.5 (24.5–49.25)
Female sex, *N* (%)	25 (66%)
Disease duration, median (IQR), months	30.5 ± 49.5 (1.0–298)
HLA-B27 +, *N* (%)	18 (47%)
Diagnosis, (%): axSpA PsA IBD-SpA	26 (68%) 9 (24%) 3 (8%)
SI, *N* (%): Unilateral Bilateral	19 (50%) 19 (50%)
SIJ erosions, *N* (%)	11 (29%)
Disease subset, *N* (%): axial disease axial + peripheral disease axial + entheseal disease	23 (60%) 9 (24%) 6 (16%)
ESR, median (IQR), mm/h	20.5 (12.0–37.0)
CRP median (IQR), mg/dl	0.35 (0.2–0.9)
Non-eligibility, *N* (%): Tumor Comorbidity Patients’ preferral	7 (18%) 11 (29%) 20 (53%)

IQR, interquartile range; *N*, number; HLA-B27, human leukocyte antigen -B27; axSpA, axial spondyloarthritis; PsA, psoriatic arthritis; IBD-SpA, inflammatory bowel disease-spondyloarthritis; SI, sacroiliitis; SIJ, sacroiliac joint; ESR, erythrocyte sedimentation rate; CRP, C-reactive protein.

Baseline median ESR value was 20.5 mm/h (12.0–37.0) and median CRP serum level was 0.35 mg/dL (0.2–0.9).

**Figure 1:**
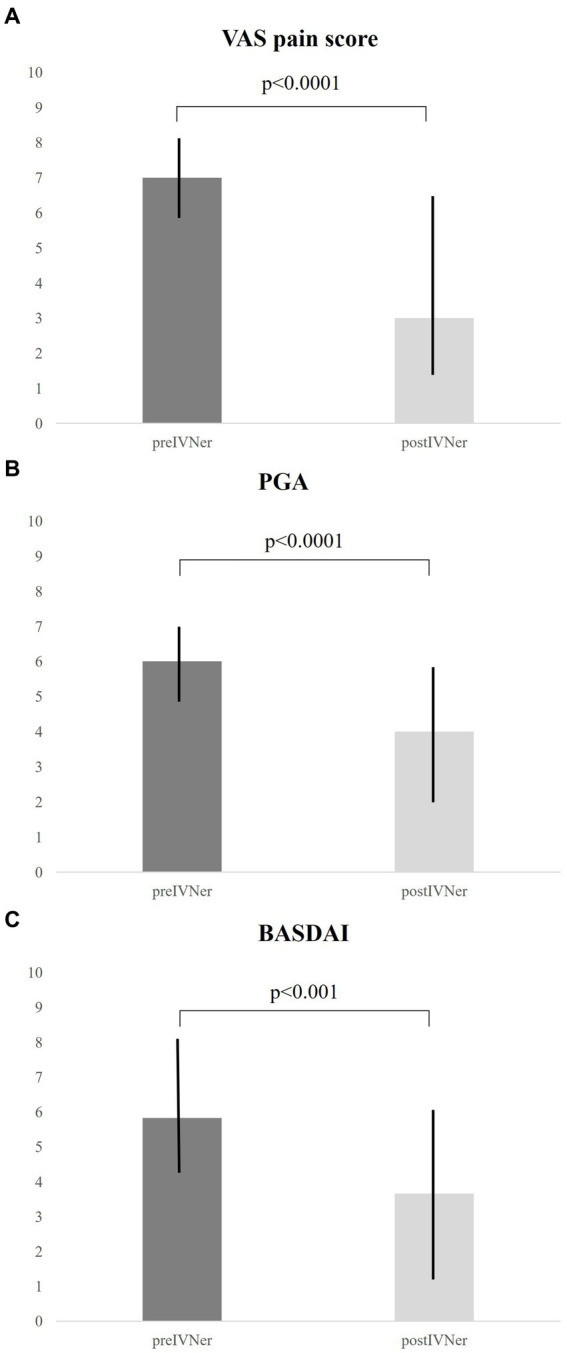
Response to IVNer was evaluated after 60 days from the last infusion as the mean changes from baseline of VAS pain **(A)**, Patient Global Assessment **(B)**, and Bath Ankylosing Spondylitis Disease Activity Index (BASDAI) scores **(C)**.

After 60 days from IVNer, median VAS pain score was significantly reduced compared to baseline [7 (5.75–8.0) vs. 3 (1.0–7.0), *p* < 0.0001, Figure 1A]. Accordingly, a significant improvement of median Patient Global Assessment (PGA) score [6 (5.0–7.0) vs. 4 (2.0–6.0), p < 0.0001, Figure 1B] and median BASDAI score [5.83 (4.2–8.33) vs. 3.66 (1.1–6.85), respectively, *p* < 0.001, Figure 1C] were observed.

Median ESR levels [16.5 mm/h (8.5–25.0) vs. 20.5 mm/h (12.0–37.0)] and CRP levels [0.3 mg/dL (0.19–0.65) vs. 0.35 mg/dL (0.2–0.9)] did not significantly vary after treatment.

Bivariate analysis showed no significant differences in VAS pain score, PGA score, and BASDAI score for age, sex, disease duration, ESR and CRP serum levels, HLAB27 positivity, unilateral SIJ involvement, and erosions at the SI level on MRI.

On day 60 after the last IVNer, many patients showed an improvement of sacroiliitis on MRI: ten (36%) patients had a complete resolution of BME, eleven (39%) patients presented a partial resolution, and seven (25%) patients showed no changes.

Compared to older age, younger patients showed a significant MRI improvement (CI 0.726–0.997, *p* = 0.045). Disease duration, sex, and HLA-B27 positivity were not the predictors of MRI improvement.

Among other explored variables (i.e., age at treatment, disease duration, HLA-B27 positivity, and female sex) at logistic regression, no predictors of treatment success were found.

## 4 Discussion

Our study demonstrated that IVNer was effective in reducing disease activity, as measured by the BASDAI score, and in reducing pain, as measured by VAS pain score in patients with axial involvement in SpA refractory to NSAIDs and untreatable with bDMARDs. Moreover, in most patients, IVNer reduced the extension of BME at SIJ on MRI.

For the first time, our study evaluated the use of BPs as a therapeutic option for patients who cannot take bDMARDs due to relative or absolute contraindications, which can be a common condition in real-life settings. Providing evidence concerning a possible treatment option could improve the management of difficult clinical situations and cover a clinical unmet need in SpA management.

ASAS-EULAR recommendation for SpA (4) provide several guidelines for different clinical settings; however, they do not provide any suggestions for patients who have relative or absolute contraindications to bDMARDs or those who do not want to undergo bDMARDs treatment.

This challenging situation is not so rare in real-life settings.

The risk of developing comorbidities is higher in patients with SpA compared to the general population (17), as clearly demonstrated by the COMOSPA study (5). In the latter study, the overall prevalence of malignancies was 3.0% (95% CI 2.46 to 3.52), with cervical cancer being the most prevalent (1.2, 95% CI 0.3 to 1.7) (5). In another national-based study population of 1,168 patients affected by SpA, 3.3% experienced malignant tumors and 4.7% had infections (6). Recent diagnosis (less than 5 years) of solid tumors temporarily contraindicates therapy with bDMARDs; in this case, BPs could be considered a safer option compared to bDMARDs.

Similarly, infections can frequently limit the prescription of bDMARDs. A meta-analysis of randomized placebo-controlled trials showed a risk of serious infection between 0 and 2.9% in patients with SpA exposed to anti-TNF (18). Patients with rheumatic diseases should be preferably vaccinated before the planned introduction of immunosuppressant agents, according to specific recommendations for vaccine (19). Despite these recommendations, the COMOSPA study demonstrated a non-optimal rate of vaccination (5). In this clinical scenario, the use of BPs could be an alternative option when bDMARDs are contraindicated for infective comorbidities.

A competent person is entitled to refuse what a doctor proposes because each person’s approach to health is a personal choice. Sometimes, doctors face the challenge of patients refusing treatment; their refusals present clinicians with more complex challenges, in both law and ethics (20). Several studies in different clinical settings explored the reasons for patients’ denial, including age, education level (21), and cultural or religious factors (20). After ensuring that patients are adequately informed and freely chosen to refuse the recommended treatment, clinicians should consider alternative options to address patients’ healthcare needs that the patients might find acceptable. In this setting, BPs have been a better accepted therapeutical option for patients refusing bDMARDs, even if these drugs are not licensed for axial SpA.

Literature provided data on the efficacy of BPs in the treatment of SpA compared to both NSAIDs and bDMARDs (9, 22, 23).

Intravenous pamidronate administered at a monthly dose of 60 mg was able to significantly reduce the mean BASDAI score by 2.22 compared to 0.93 in the group receiving a monthly dose of 10 mg, and the same decrease was reported for Bath AS Functional Index (*p* < 0.001), Bath AS Global Index (*p* = 0.01), and Bath AS Metrology Index (*p* = 0.03) (9).

In a 48-week open randomized trial, efficacy of 60 mg IV pamidronate 4 weekly for 48 weeks was compared to 50 mg monthly golimumab. After 48 weeks, there was not significant differences in the two arms in terms of ASAS20 response (56% vs. 65%; *p* = 0.69), with an overlapping safety profile (22).

Viapiana and colleagues explored the efficacy profile of monthly IVNer (100 mg) versus standard infliximab (5 mg/kg) therapy in a 6-month open-label study on active AS showing a significant reduction in the mean BASDAI of either IVNer (− 1.72) or infliximab (−1.62) administration (23).

The efficacy of BPs relies not only on their antiresorptive capacity; in fact, BPs demonstrated to be able to reduce the production of several pro-inflammatory cytokines, such as TNF-*α*, IL-6, and IL-1 (8). In recent times, the mechanism of bone involvement in axSpA is only partially understood. Indeed, axSpA is characterized by the coexistence of local bone formation and bone resorption predominantly at the SIJ and the spine. Inflammation results in erosions of the SIJ, and prolonged severe manifestation lead to ankylosis of the SIJ and the spine. The dual effect on bone in SpA makes the interpretation of markers of bone formation and bone degradation very complex. However, a few studies highlighted that bone resorption markers are increased in active ankylosing spondylitis and are associated with changes in BMD (24). In this setting, the action of IVNer on osteoclasts can slow down their activity. Furthermore, increasing evidence highlighted monocytes and macrophages as direct targets of BP action, which may explain the acute phase response and the anti-tumor activity in certain animal models (25).

Another possible explanation on BPs effect could rely on their ability to reduce circulating γδ lymphocytes (26), modulating anti-inflammatory and pro-inflammatory cytokines derived from Th17 and Treg populations (14), as demonstrated in patients treated with chronic N-BPs for osteoporosis.

The anti-inflammatory properties of BPs could explain the significant reduction in disease activity scores, as demonstrated by BASDAI reduction from baseline to 60 days after the last IVNer infusion.

Furthermore, our study demonstrated that a shorter interval of “higher” regimen of IVNer showed rapid improvement on clinimetric parameters (24 weeks and 48 weeks versus 8 weeks since treatment start). This could probably be related to the higher drug local concentration that can be achieved with a shorter interval of N-BPs administration in proper dosages.

IVNer demonstrated to be effective in reducing pain expressed as a decrease of VAS pain score. In literature, BPs proved to reduce the expression of neuromediators on animal models of complex regional pain syndrome type 1. In fact, BPs significantly reduced tissue expression of nerve growth factor (13). The effect on pain score reduction can be mediated by BP action on neuroflogosis mediators. Furthermore, in our cohort, we had a median of 30.5 ± 49.5 months, indicating that some patients had a long-standing disease before starting IVNer. In a long-standing disease, mechanisms of pain dysregulation, such as fibromyalgia, may be associated with SpA; in fact, we reported 16% of patients with fibromialgya associated with SpA.

IVNer demonstrated to reduce BME associated with active SI. BPs showed more promising results in the so-called “Primary Bone Marrow Edema Syndromes” (27). Although there is no ultimate demonstration of efficacy and a shared approval yet, data from open studies and case reports suggest that the treatment is highly effective in curing diseases such as BME of the hip and transient regional osteoporosis (28), with fast pain resolution and improvement of radiological and densitometric findings. The more convincing results are derived from RCTs on BP treatment of CRPS-1 (29).

Our data demonstrated that IVNer could induce a resolution of BME, mainly in younger patients compared to older ones. Several questions have been raised about the specificity of BME signal and active SI because these findings could be incidentally found in heathy individuals or athletes or could be related to degenerative diseases. Several reports highlighted that BME related to the degenerative disease is located at the upper and anterior part of the joint, where biomechanical loads are prevalent (30). Another recent report explored the association between BME (defined as SI ASAS) and non-inflammatory spine abnormalities in patients suffering from chronic mechanical back pain. The study revealed that BME at the lower and posterior parts of the SIJ is not indicative of an inflammatory condition. Therefore, it is possible to speculate that incomplete resolution of BME in older individuals could be related to a mechanical/postural component, which is undeletable, indicating the coexistence of inflammatory and degenerative conditions.

Our study has some strengths. First, we address a possible alternative therapy in active SpA when relative or absolute contraindications to bDMARDs exist. Second, our study demonstrated that a shorter interval of “higher” regimen of IVNer showed the rapid improvement on clinimetric parameters.

Our study also has several limitations. The retrospective design requires a degree of caution. Furthermore, we evaluated VAS pain, PGA, and BASDAI scores that are all outcomes based on patients’ subjective evaluation; however, we also used few objective outcome measures such as MRI. Third, the power of our study was not comparable to previous RCTs and we had missing data in a small cohort that prevents definitive conclusions, particularly on long-term radiological evolution.

## 5 Conclusion

N-BPs cannot be regarded as being equivalent to DMARDs in terms of efficacy. However, their efficacy profile may be considered in particular clinical settings, when absolute or relative contraindication to preferable therapies exists, given also their low costs and good safety profile. Therefore, further RCTs should be designed to address their potential contribution in SpA damage prevention, both on the inhibition of syndesmophytes formation and other SpA-related issues, such as the prevention of vertebral fractures.

## Data availability statement

The raw data supporting the conclusions of this article will be made available by the authors, without undue reservation.

## Ethics statement

The studies involving humans were approved by the Comitato Etico Istituto Ortopedico G. Pini. The studies were conducted in accordance with the local legislation and institutional requirements. The participants provided their written informed consent to participate in this study.

## Author contributions

CC: Conceptualization, Data curation, Formal analysis, Investigation, Methodology, Project administration, Supervision, Writing – original draft. RT: Data curation, Formal analysis, Investigation, Writing – original draft. FO: Data curation, Formal analysis, Investigation, Writing – original draft. MF: Data curation, Formal analysis, Investigation, Writing – original draft. MV: Supervision, Writing – review & editing. EGF: Supervision, Writing – review & editing. RC: Supervision, Writing – review & editing.
